# The Diverse Function of PD-1/PD-L Pathway Beyond Cancer

**DOI:** 10.3389/fimmu.2019.02298

**Published:** 2019-10-04

**Authors:** Weiting Qin, Lipeng Hu, Xueli Zhang, Shuheng Jiang, Jun Li, Zhigang Zhang, Xu Wang

**Affiliations:** State Key Laboratory of Oncogenes and Related Genes, Shanghai Cancer Institute, Ren Ji Hospital, School of Medicine, Shanghai Jiao Tong University, Shanghai, China

**Keywords:** PD-1, PD-L1, autoimmune, chronic infection, sepsis

## Abstract

The recent success of PD-1 and PD-L1 blockade in cancer therapy illustrates the important role of the PD-1/PD-L1 pathway in the regulation of antitumor immune responses. However, signaling regulated by the PD-1/PD-L pathway is also associated with substantial inflammatory effects that can resemble those in autoimmune responses, chronic infection, and sepsis, consistent with the role of this pathway in balancing protective immunity and immunopathology, as well as in homeostasis and tolerance. Targeting PD-1/PD-L1 to treat cancer has shown benefits in many patients, suggesting a promising opportunity to target this pathway in autoimmune and inflammatory disorders. Here, we systematically evaluate the diverse biological functions of the PD-1/PD-L pathway in immune-mediated diseases and the relevant mechanisms that control these immune reactions.

## Introduction

Immune checkpoint inhibitory receptors [such as cytotoxic T lymphocyte antigen 4 (CTLA4) and programmed cell death protein 1 (PD-1)] expressed on immune cells trigger immunosuppressive signaling pathways. CTLA4 competes with CD28 for binding to CD80 and CD86 (1). PD-1 binds to PD-L1 or PD-L2 and resists positive signals through T-cell receptors (TCRs) and CD28 (2). These immunosuppressive molecules function as brakes to regulate the adaptive immune response. To date, suppressive signals have been used in a variety of ways to maintain the balance of the immune system. Their use has also been translated to the clinic. Recent years have witnessed the wide application of anti-PD-1, anti-CTLA4, and anti-PD-L1 antibodies in various types of cancer (3). Based on this application, the PD-1/PD-L pathway has stood out as an immune checkpoint.

In general, expression of PD-L1 is observed on T, B, and antigen-presenting cells and in some non-lymphoid tissues. Ligand binding to PD-1 on the surface of T cells mediates immune inhibition. In addition, PD-L1 is detected in the cardiac endothelium, placenta, and pancreatic islets, which indicates a role of PD-L1 in immunological tolerance (4). PD-L2 is another ligand of PD-1. The function of PD-L2 is not as well-known as that of PD-L1; thus, its clinical utility is still being explored. It has been reported that PD-L2 plays an inhibitory role similar to that of PD-L1 (5). In addition, it has been shown that PD-L2 plays an opposing role to PD-L1 in T-cell function in a PD-1-independent manner (6).

Recent reviews have extensively discussed the role and application of the PD-1/PD-L1 pathway in cancer. Recent studies have shown that inhibitory pathways are also involved in immune evasion by pathogens, suggesting that PD-1/PD-L may play a vital role in infections (7). In addition, studies using animal models of autoimmune diseases have shown that the interaction between PD-1 and PD-Ls is pivotal for the regulation of peripheral tolerance and autoimmunity (8). These data suggest that beyond their role in cancer, PD-1 and PD-Ls can be good candidates for the treatment of these diseases. However, whether this targeting could succeed may need to be further explored.

Here, we systematically evaluate the diverse biological functions of PD-1/PD-L in diseases other than cancer, including autoimmunity, chronic infections, and sepsis. For each disease, we cover the current understanding of the roles that PD-1 and PD-Ls play and the relevant mechanisms that control these immune reactions.

## PD-1/PD-L Axis

**Figure 1:**
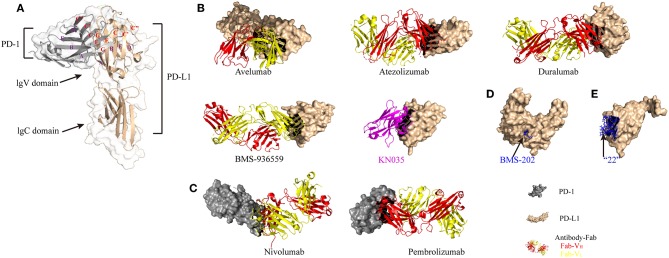
Structural basis of PD-1/PD-L1 checkpoint blockade by antibodies and small molecules. **(A)** Structure of the PD-1 and PD-L1 complex (modified from PDB ID 3BIK). **(B)** Overall structure of the hPD-L1 and avelumab-Fab complex, the hPD-L1 and atezolizumab-Fab complex, the hPD-L1 and durvalumab-Fab complex, the hPD-L1 and BMS-936559-Fab complex, and the hPD-L1 and KN035 nanobody complex (magenta ribbon model) (modified from PDB IDs 5GRJ, 5X8L, 5X8M, 5GG7, and 5JDS, respectively). The heavy chain (V_H_) and the light chain (V_L_) of the antibody-Fab fragments are represented by red and yellow ribbon models, respectively. **(C)** Overall structure of the hPD-1 and nivolumab-Fab complex and the hPD-1 and pembrolizumab-Fab complex (modified from PDB IDs 5GGR and 5GGS, respectively). **(D)** Crystal structure of BMS-202 (non-peptide small molecule inhibitor, blue stick model) on the surface of hPD-L1 (light yellow ribbon structure) (modified from PDB ID 5J89). **(E)** Structure of macrocyclic peptide inhibitor “22” in complex with hPD-L1 (light yellow ribbon model = hPD-L1; blue ball and stick model = peptide) (modified from PDB ID 5O45).

PD-1 (CD279), a member of the CD28/CTLA4 family, is a receptor for PD-L1 and PD-L2 (1, 2, 5). PD-L1 (CD274) and PD-L2 (CD273) are both B7 family members, sharing 60% amino acid homology in humans and 77% amino acid homology in mice (9). Moreover, there is high diversity in the structure of PD-1 between humans and mice. Binding of PD-L1 to PD-1 has been found to exhibit similar affinity in both species (humans and mice) *in vitro*. Recently, the structures of mouse (m) PD-1/human (h) PD-L1, mPD-1/mPD-L2, and hPD-1/hPD-L1 have been reported, which may promote protein affinity engineering (10). With the breakthrough in the structural elucidation of the PD-1/PD-L1 complex, targeting the PD-1/PD-L1 immunological checkpoint with monoclonal antibodies and small molecular drugs has seen great progress (Figure 1). To date, five antibody-based inhibitors targeting PD-1/PD-L1 have been approved by the Food and Drug Administration (FDA), including two anti-PD-1 antibodies (nivolumab and pembrolizumab) and three anti-PD-L1 antibodies (avelumab, atezolizumab, and durvalumab). Nivolumab has been approved for non-small-cell lung cancer (NSCLC), renal cell carcinoma (RCC), bladder cancer (BC), colorectal cancer (CRC) with microsatellite instability or mismatch repair deficiency (MSI-H/dMMR), hepatocellular carcinoma (HCC), classic Hodgkin lymphoma (cHL), melanoma, and head and neck squamous cell carcinoma (HNSCC). Pembrolizumab has been approved for melanoma, HNSCC, cervical cancer, cHL, NSCLC, BC, stomach and gastroesophageal cancers, and all advanced solid tumors classified as MSI-H/dMMR. Avelumab has been approved for Merkel cell carcinoma and BC. Atezolizumab has been approved for NSCLC and BC. Durvalumab has been approved for BC and NSCLC (stage III) (11). In addition, an experimental anti-PD-L1 antibody (BMS-936559) and a nanobody targeting PD-L1 (KN035) have been developed as potential new drugs (12). KN035 is being evaluated in phase I clinical trials in patients with advanced solid tumors. Furthermore, several promising small molecules, including non-peptide and peptide-based molecules, are also designed to target this immune checkpoint pathway [see (13) for detailed information on small-molecule inhibitors]. For instance, a novel small-molecule inhibitor named “22” can inhibit the interaction between PD-1 and PD-L1 *via* binding at the PD-1–PD-L1 interface (14).

PD-1 and its ligands are transmembrane proteins and include an extracellular domain, a transmembrane region, and a cytoplasmic tail. PD-L1 is a type I transmembrane glycoprotein composed of typical IgC and IgV domains. Figure 1A shows a 1:1 receptor/ligand stoichiometry, with interactions primarily between the faces of the IgV domains GFCC' β sheet (indicated by the red letters). Notably, ligands of PD-1 with a short cytoplasmic tail may not transduce signaling through this pathway. Blockade of TCR and costimulatory signals is the primary effect of PD-1/PD-L1 signaling. Like in TCR signaling, CD28 may be the key target (15–17). Signaling through PD-1 is well-understood [see (4, 18) for detailed information on the PD-1/PD-L1 pathway], while there are fewer studies focusing on PD-L1 signaling. It has been reported that in the intracellular domain of PD-L1, there is a specific site that can be phosphorylated. This site shares 16% homology with B7-2 (CD86), which has been shown to transduce reverse signaling *via* cross-linking with mAbs. Furthermore, *in vitro* treatment with PD-L1 agonists (PD-L1 mAbs or PD-1 Ig) in Epstein-Barr virus (EBV)-transformed B cells can trigger cell apoptosis *via* cross-linking of PD-L1 (19). The above studies suggest that reverse signaling through PD-L1 exists, and further research should be conducted on this process.

## PD-1/PD-L Axis Expression

Expression of PD-1 can be observed on immature CD4^−^CD8^−^ thymocytes, a small fraction of murine thymocytes, lymph node, spleen, and bone marrow cells [for a review, see (20)]. The level of PD-1 (mRNA or protein) is rarely detectable, only becoming evident after a period of stimulation. Several factors can induce PD-1 expression. Generally, in lymphocytes, cytokines and the factors that stimulate B-cell receptor (BCR) or TCR signaling [such as Con A, phorbol 12-myristate-13-acetate (PMA)/ionomycin, and anti-IgM antibody] have the capacity to promote the expression of PD-1 (21, 22). Notably, it was reported that estrogen stimulation can also promote PD-1 expression on T lymphocytes and antigen-presenting cells (APCs) (23). Recently, it has been found that in mouse cytomegalovirus infection, endogenous glucocorticoids can induce selective and tissue-specific expression of PD-1 on NK cells (24), suggesting that NK cells are potential PD-1 blockade responders.

Currently, PD-L1 is expressed not only on hematopoietic cells [T cells, B cells, macrophages, dendritic cells (DCs), and neutrophils] but also in some non-hematopoietic tissues (heart, pancreas, placenta, vascular endothelium, muscle, liver, lung, eye, and skin tissues). PD-L1 expression in normal tissues suggests that the PD-1/PD-L1 signaling pathway may prevent tissue inflammation and contribute to homeostasis maintenance [for reviews, see (4, 25)]. However, PD-L1 expression can also be observed in cancers and plays a critical role in the avoidance of immune surveillance by cancerous cells. Unlike PD-L1 expression, PD-L2 expression is mainly restricted to APCs (26). Given the importance of the PD-1/PD-L1 axis, it is interesting to explore the regulatory mechanisms underlying its components.

PD-L1 is located on chromosome 9p24.1 and is only 42 KB from PD-L2. It has been implied that the elevated PD-L1 level is associated with amplification and translocation of this gene. In cases of NSCLC, SCLC, Hodgkin lymphoma, mediastinal large B-cell lymphoma, squamous cell carcinoma of the oral cavity, and Epstein-Barr virus-positive gastric cancer, amplification of the chromosomal portion containing PD-L1 has been reported [for a review, see (16)]. In addition, inflammatory signaling has been reported to modulate PD-L1 levels. Over the past few years, various soluble factors produced by immune cells have been described as inducers of PD-L1. Interferon IFN-γ has been considered one of the most potent inducers of PD-L1 expression. Importantly, the regulation of PD-L1 expression by IFN-γ is different between tumor and immune cells. For instance, in a mouse model of sarcoma, with IFN-γ blockade, the expression of PD-L1 was greatly decreased on tumor cells but only partially on tumor-associated macrophages (TAMs), which indicated that the expression of PD-L1 on TAMs is partially regulated by IFN-γ (27). This context-dependent effect on PD-L1 expression was also observed for transforming growth factor (TGF)-β and interleukin (IL)-12. Specifically, *in vitro* treatment of cultured monocytes and tubular epithelial cells with TGF-β inhibited PD-L1 expression (28). In a pancreatic islet transplantation model, TGF-β was required for sustained expression of PD-L1 on monocytes and tubular epithelial cells (29). Similarly, IL-12 promotes PD-L1 expression in endothelial cells and monocyte-derived macrophages but negatively regulates PD-L1 levels in THP-1 cell line-derived macrophages (30). In addition, other stimulators have been shown to induce PD-L1 expression [for review, see (16, 18)]. Monocytes and macrophages, as well as T cells, express elevated levels of PD-L1 when stimulated with IL-2, IL-17, and IL-15. IL-4 and granulocyte-macrophage colony-stimulating factor (GM-CSF) upregulate PD-L1 in macrophages. DCs express increased levels of PD-L1 when treated with IL-1β, IL-6, IL-10, and IL-27. Tumor necrosis factor (TNF)-α induces PD-L1 expression in dendritic cells, monocytes, endothelial cells, myelodysplastic syndrome blast cells, RCC cells, prostate cells, breast cancer cells, and colon cancer cells. Lipopolysaccharide (LPS) increases PD-L1 expression in macrophages, monocytes, DCs, neutrophils, and B cells. Poly(I:C) upregulates PD-L1 expression in lung cells.

Notably, the expression of PD-L1 can also be controlled by microRNAs, which regulate the *PD-L1* gene by binding to its 3 prime untranslated region (3′UTR). The reported microRNAs include miR-155, miR-513, miR-34a, miR-142-5p, miR-93, miR-106b, miR-138-5p, miR-200, miR-152, miR-570, miR-17-5p, miR-15a, miR-193a, miR-16 [for a review, see (16)], miR-148a-3p, and miR-873 (31, 32). Consistent with this finding, deletion of the 3′UTR of the *PD-L1* gene enhances PD-L1 mRNA stability in human and murine cells (33). In addition, cells that express high PD-L1 levels are observed to have loss of the 3′UTR in the *PD-L1* gene, which is reported to result from gene trap vector integration (34). MicroRNAs (such as miR-20, miR-21, miR-130b, miR-197, and miR-223) can also regulate the expression of PD-L1 in an indirect way (35, 36). For example, mDCs from miR-223-KO mice display elevated PD-L1 expression mainly *via* regulation of the expression of STAT1, which is a crucial regulator of PD-L1 expression. Notably, the PD-L1 protein can be glycosylated, leading to inaccurate immunohistochemical readouts resulting from the inaccessibility of PD-L1 antibodies to PD-L1 polypeptide antigens. Lee et al. (37) found that deglycosylation can significantly ameliorate the above problem and provide an effective approach to guide anti-PD-1/PD-L1 therapy.

## PD-1/PD-L Axis Function

Although the PD-L2 binds to PD-1 with higher affinity than does PD-L1, the primary ligand of PD-1 is PD-L1, as supported by the studies in PD-L1 knockout mice. The relevant mechanism is still unknown. As mentioned above, both PD-1 and its ligands are transmembrane proteins that include an extracellular domain, a transmembrane region, and a cytoplasmic tail. Upon binding of PD-L1, PD-1 activation leads to phosphorylation of the immunoreceptor tyrosine-based switch motif (ITSM) in the cytoplasmic tail and recruitment of Src homology region 2 domain containing phosphatase 1/2 (SHP1/2) and the newly identified slam-associated protein (SAP) (SH2D1A) (38). Then, through PD-1/PD-L1 signaling, TCR (such as ZAP 70) and costimulatory (such as PI3K/AKT) signaling cascades are inhibited (39). Moreover, as reported, like in TCR signaling, CD28 may be the key target. In detail, SHP1/2 is recruited to the ITSM, leading to dephosphorylation of the TCR and CD28 proximal signaling molecules (e.g., ZAP70, PKCθ, and other kinases) (40). Then, the inhibitory signaling contributes to the inactivation of downstream signaling pathways, such as the PI3K/AKT and Ras-MEK-ERK pathways. Additionally, SHP-2 can dephosphorylate casein kinase 2 (CK-2), leading to PTEN activation, in turn suppressing PI3K/AKT signaling (41). SHP-2 targets phospholipase Cγ and can inhibit the Ras-MEK-ERK cascade (42).

Binding of PD-L1 to PD-1 on different cells contributes to different functions. For instance, in effector T cells expressing PD-1, PD-L1 binding suppresses TCR or costimulatory signaling, resulting in T-cell apoptosis, anergy, and exhaustion (2, 43–46). In innate lymphoid cells (ILC2s), PD-L1 binding to the PD-1 receptor dampens ILC2 proliferation and function (47). However, PD-L1 binding to PD-1 on regulatory T (Treg) cells leads to their proliferation. Moreover, PD-L1 binding to PD-1 on naive T cells results in polarization of CD4^+^ T cells toward the T helper type 1 (TH1) subtype with effector T (Teff) cell proliferation [for review, see (16, 48)]. In follicular T helper (Tfh) cells expressing PD-1, PD-L1 binding regulates Tfh cell recruitment, restricts their localization to the germinal center (GC), and maintains the stringency of affinity selection in the GC (49). In some settings, PD-1 can protect cells from T-cell attack by shortening the interaction time between cytotoxic T lymphocytes (CTLs) and target cells (29). Therefore, PD-1/PD-L1 signaling plays a vital role in immune tolerance and T-cell exhaustion and has emerged as a key target in the treatment of cancer.

Interestingly, in some settings, the promotion of inflammation by PD-L1 and PD-L2 does not require PD-1 expression on target cells, suggesting the existence of a currently unknown proinflammatory receptor for PD-L1 and PD-L2. Wang et al. (50) found that PD-L1 signals through receptors that remain unidentified to activate T cells. B7-1 (CD80) cross-linking activates CD4^+^ T cells and increases the production of proinflammatory cytokines by T cells (50). Notably, another form of PD-L1, soluble PD-L1 (sPD-l1), has been identified. *Via* matrix metalloproteinase activity, sPD-L1 is released from the cell surface (51); therefore, the level of sPD-L1 indirectly accounts for the PD-L1 level, and it has been suggested that PD-1 interaction with PD-L1 may be blocked by sPD-L1.

The above findings illustrate the PD-1/PD-L1 pathway. However, the role of PD-L2 in regulating the immune response is still unclear. In general, on the one hand, interaction of PD-L2 with PD-1 may inhibit the activation of T cells (5). On the other hand, PD-L2, in a PD-1-independent manner, plays an antitumor role, inducing the production of T_H_1 cytokines and CTLs (6). Notably, it has been shown that an aggregated form of PD-L2 can suppress the interaction between PD-1 and PD-L1, which was observed on DCs (52).

Above, we reviewed the fundamental biology of the PD-1/PD-L immune checkpoint. In the following sections, the therapeutic potential of the PD-1/PD-L axis in cancers and other disorders (focusing on autoimmunity, chronic infection, and sepsis) will be thoroughly discussed.

## Cancer

PD-L1 and/or PD-L2 expression either in tumor or in infiltrating immune cells has been verified in numerous tumors, indicating a role for the PD-1/PD-L1 axis as a prognostic trait and therapeutic target. Researchers have found that PD-1 expression on tumor-infiltrating lymphocytes (TILs) correlates with aggressive features (8, 53) and is linked to poor patient outcome. Furthermore, transgenic expression of PD-L1 in mouse tumor cell lines (such as melanoma, mastocytoma, myeloma/plasmocytomas) has additional escape ability from the host T cells and enhances the invasiveness *in vivo* (8). Till now, the regulation of PD-L1 has been well–researched, and it involves many pathways [see (54, 55) for detailed information on PD-L1-expressing regulation]. Studies show that the oncogenic signaling could regulate the expression of PD-L1 in tumor. For instance, in anaplastic lymphoma kinase (ALK)-carrying T-cell lymphoma, PD-L1 expression can be induced by ALK signal transducer, and this function is also observed in signal transducer and activator of transcription 3 (STAT3) in chemoresistant NSCLC (35). Besides, IFNs, especially IFN γ, and some other stimuli [e.g., IL-4, IL-10, vascular endothelial growth factor (VEGF), G-CSF, and LPS] are involved in PD-L1 regulation (56).

It has been demonstrated that PD-L1 protects cancer cells from direct attack by cytotoxic T cells. Binding of cancer cells expressing PD-L1 on their surface to PD-1 expressed on activated CD8^+^ T cells results in anergy and apoptosis of CD8^+^ T cells (57). It has been reported that thymocyte selection-associated high-mobility group box (TOX) expression coincides with PD-1 expression and can reinforce the phenotype and longevity of these exhausted T cells (58, 59). Interestingly, PD-L1 expressed on cancer cells can also serve as an antiapoptotic factor, leading to resistance to lysis by CD8^+^ T cells (60). Thus, cancer can escape from immune system attack *via* the PD-1/PD-L1 pathway, which suggests that blockade of this pathway may generate valuable results. The FDA approved two anti-PD-1 antibodies (pembrolizumab and nivolumab) in 2014, one anti-PD-L1 antibody (atezolizumab) in May 2016, and two PD-L1 antibodies (durvalumab and avelumab) in 2017 (61). The clinical success of these therapies has been shown in patients with melanoma, metastatic lung cancer, kidney cancer, BC, head and neck cancer, urothelial carcinoma, hepatocellular carcinoma, gastric cancer, metastatic Merkel cell carcinoma, and Hodgkin lymphoma (62). Certainly, it is unfeasible to attain stable and long-term disease remission with a single agent. In addition, a proportion of patients fails to respond to these therapies. The next wave in the treatment of cancer will focus on combination therapies (63, 64). Currently, the combination of PD-1 inhibitors with either CTLA-4 blockade or chemotherapy has been approved by the FDA and has shown improved clinical response rates compared with the single agents (65, 66).

Importantly, the interplay between the tumor and the immune system is complex, and both entities affect the outcome. In general, tumor foreignness, patient health status, other inhibitory processes within the tumor, functional exhaustion of tumor-infiltrating lymphocytes, and the sensitivity of tumor cells to tumor-specific T cells may affect the outcome of PD-1/PD-L1 blockade. Some other strategies for combination therapies with PD-1/PD-L1 blockade are being developed, including blockade of other inhibitory receptors and immunoregulatory cytokines, administration of agonists of costimulatory molecules, and treatment with homeostatic cytokines or engineered T cells [see (39) for detailed information on combination strategies]. More recently, Li et al. (67) showed that B7S1-B7S1R signaling additionally regulates CD8^+^ T-cell responses by cooperating with PD-1/PD-L1 checkpoint signaling, which emphasizes the importance of improving the understanding of T-cell checkpoint signaling and strategies to maximally and intelligently employ these therapeutic approaches alone or in combination.

## Autoimmunity

Autoimmune diseases are defined as aberrant immune responses of an organism to its own cells and tissues. The incidence of autoimmunity is increasing worldwide, affecting ~5% of the population (68). The immunological abnormalities can precede autoimmune diseases by months to years (69). Autoimmunity can result from the escape of antigen-specific autoreactive T cells from the thymus to the periphery in the perinatal period (70).

PD-1 and PD-Ls have been demonstrated to be involved in the modulation of both central and peripheral tolerance. In particular, during thymocyte development, PD-1 plays a critical role by regulating signaling thresholds during positive selection (71). Consequently, the population of CD4^+^CD8^+^ thymocytes is increased in the absence of PD-1 or PD-L1 (72). Moreover, the PD-1 pathway serves as a negative regulator in autoreactive T and B cells to maintain tolerance (73).

In the setting of autoimmunity, PD-1 and PD-L1 have been studied in PD-1 knockout (PD-1^−/−^) mice, where breakdown of peripheral tolerance results in negative regulation of lymphocyte activation. PD-1 knockout can lead to different autoimmune features depending on the genetic background (44). PD-1 knockout mice in the BALB/c background, C57BL/6 background, and non-obese diabetic (NOD) background may develop dilated cardiomyopathies, lupus-like diseases, and type 1 diabetes mellitus (T1DM), respectively. Since the genetic background is a key factor in PD-1/PD-L signaling, it is important to elucidate the specific role of this axis in humans. Notably, the role of PD-L1 and PD-L2 in susceptibility regulation and autoimmune response progression may be different. For example, PD-1 and PD-L1 knockout mice showed more severe experimental autoimmune encephalomyelitis (EAE) symptoms than PD-L2 knockout or control mice (74). Furthermore, in some experimental animal models (diabetes, EAE, and autoimmune enteritis), administration of anti-PD-1/PD-L1 antibodies accelerates autoimmunity (75–77). Indeed, administration of a PD-L1 Ig recombinant fusion protein, PD-1-specific toxins [αPD-1-ABD-PE: anti-PD-1 single-chain variable fragment (αPD-1), an albumin-binding domain (ABD) and *Pseudomonas* exotoxin (PE)], or adenovirus expressing Fc-PD-L1 can reduce the severity of inflammatory diseases [rheumatoid arthritis (RA), colitis, lupus-like nephritis, EAE, systemic lupus erythematosus (SLE) and psoriasis] (Table 1) (78–80, 94, 95). In addition, upregulation of PD-1/PD-L1 expression can decrease disease onset and severity (autoimmune diabetes and EAE) (96). Furthermore, cells (such as umbilical cord mesenchymal stem cells and tonsil/bone mesenchymal stem cells) expressing PD-L1, which has the potential to inhibit lymphocyte proliferation, exhibited reduced immunogenicity and improved immunosuppressive capacity, which have been reported to lead to effective immunotherapy for diseases such as psoriasis (97).

**Table 1:** Summary of studies reporting PD-1/PD-L1 in autoimmunity and inflammatory diseases.

	**Study**	**Cell type**		**Target species**	**Model**	**References**
		**PD-1**	**PD-L1**			
Autoimmunity	SLE	m: CD4^+^ and CD8^+^ T cell	h: pDC, neutrophil and macrophage	Humans and mice	Patients with SLE Lupus-prone NZB × W F1 mice Murine PD-L1-Ig was injected into SLE-prone mice SLE-prone mice treated with anti-PD-L1 antibody	(78, 79)
	Psoriasis	h/m: T cell(γδT)	h: pDC m: Gr-1^+^, CD4^+^, and CD11c^+^ cell	Humans and mice (*ex vivo*)	Person with psoriasis Imiquimod-treated mice mimic psoriasis PD-L1-Fc were administered in imiquimod-treated mice γδT cells from imiquimod–treated mice treated with PD-L1-Fc	(79, 80)
	IBD	h: CD4^+^ and CD8^+^ T cell m: DC		Humans and mice	DSS-induced acute and T-cell-induced chronic colitis models Administrated with Ad/PD-L1-Fc and rPD-L1-Fc protein in the colitis models B7-H1-deficient mice treated with DSS or TNBS CD patients	(81, 82)
Chronic infection	IVA	iNK, CD8^+^ T cell and ILC	DC and iNK	Mice	Mice were infected with IVA Ttreated with anti-PD-L1/PD-1 antibody throughout infection PD-L1/PD-L2 deficient mice infected with IVA Adoptive transfer of iNKT cells from wild type, PD-L1 or PD-L2 deficient mice into iNKT cell deficient mice	(83–85)
	Listeria		CD4, CD8^+^ T cell, B cell	Mice	Mice were infected with cfu LM-OVA After infection adminstrated with mAb specific for PD-L1, PD-L2, PD-1, or PD-L1-CD80	(86)
	Malaria	h/m: CD4^+^ T cell	m: CD4^+^ and CD8^+^ T cell	human and Mice	Malian children with malaria pRBC infected mice induce malaria After infection treated with anti-PD-L1 antibody anti-PD-L2 antibody	(87, 88)
Sepsis	CLP	CD4^+^ and CD8^+^ T cell, B cell, monocyte, iNKT, and KC	CD4^+^ and CD8^+^ T cell, B cell, monocyte, iNKT, and neutrophil	Mice (*ex vivo*)	CLP PD-L1 ^−/−^ treated with CLP CLP treated with Anti-PD-L1 antibody Adoptive transfer of PD-L1 deficient donor iNKT cells following CLP	(89, 90)
	CLP with Candida infection	NK, NKT, CD4^+^, and CD8^+^ T cell	NK, NKT, macrophage	Mice	Two-hit model of CLP followed by Candida albicans After two-hit model administered with Anti-PD-1 and anti-PD-L1 antibody	(91)
	sepsis, septic shock	CD4^+^ and CD8^+^ T cell, monocyte and NK	Monocyte, neutrophil, and CD4^+^ and CD8^+^ T cell	Humans (*ex vivo*)	Septic/septic shock patients Lymphocytes from septic patients treated with anti-PD-1 or anti-PD-L1 antibody Incubation of whole blood from septic patients with anti–PD-L1 mAb	(92, 93)

*CD, Crohn's disease; CLP, cecal ligation and puncture; DSS, dextran sodium sulfate; IBD, inflammatory bowel disease; ILC, Innate lymphoid cell; iNKT, invariant natural killer T cell; IVA, influenza virus A; KC, Kupffer cell; mDC, monocytes myeloid dendritic cell; NOD, non-obese diabetic; pDC, plasmacytoid dendritic cell; pRBC, Plasmodium infected red blood cell; SLE, systemic lupus erythematosus; TNBS, trinitrobenzenesulfonic acid. h, human; m, mice*.

The PD-1/PD-L1 pathway protects normal host tissues mainly *via* two aspects: promoting Treg development and function and directly inhibiting self-reactive T cells. The interaction between PD-1 and PD-L1 reduces antigen-specific T-cell activation, proliferation, and effector function (98). PD-1^−/−^ mice demonstrated enhanced occurrence and severity of collagen-induced arthritis, which was linked to elevated T-cell proliferation and cytokine secretion (IFN-γ and IL-17) (99). Experiments in a murine model of SLE have shown that Tregs suppress autoreactive B cells *via* the interaction of PD-1 with PD-L1 (100). In addition, in actively induced and adoptively transferred experimental uveitis, treatment of mice with interphotoreceptor retinoid binding protein (IRBP)-specific T cells preincubated with PD-L1^hi^ retinal pigment epithelial (RPE) cells, which acquired the Treg phenotype, delayed the induction of uveitis (75). Moreover, PD-1 can regulate chemokine receptor expression on T cells. Endothelial cell expression of PD-L1 may inhibit the migration of T cells to non-lymphoid tissues (101). Further studies should be performed to assess the effects of PD-1/PD-L1 signaling on T-cell migration. Additionally, the PD-1/PD-L1 pathway can regulate the progression of diseases *via* the regulation of other immune cells (DCs and NKT cells). In different murine colitis models [dextran sulfate sodium (DSS)-induced and T cell-induced colitis], administration of PD-L1-Fc reduced the Th17 cell frequency, DC function, and disease activity (Table 1) (81, 82). Consistent with this finding, PD-L1-Fc can also mitigate the pathogenesis of immune thrombocytopenia (ITP) by increasing T-cell apoptosis and by suppressing T-cell activation and proliferation and cytokine (IFN-γ, IL-2) production (102).

The widespread expression of PD-1/PD-L1 on both hematopoietic and non-hematopoietic cells indicates its pivotal role in tissue tolerance maintenance. Generally, in tissues expressing high levels of PD-1 on autoreactive T cells, PD-L1 binding regulates autoreactive T cells. Islet β-cells express high levels of PD-L1 and can suppress autoreactive T cells. Recently, in a Japanese cohort of autoimmune diabetic patients (type 1A), CD4^+^ T cells expressed lower levels of PD-1 than those of patients with type 1 (fulminant type 1) or type 2 diabetes or healthy control subjects, which may indicate that CD4^+^ T cells with decreased PD-1 expression result in Type 1A autoimmune diabetes *via* T-cell activation (103). Consistent with this finding, low levels of PD-1 expression led to active disease in SLE patients. Interestingly, in multiple sclerosis (MS) lesions, the PD-L1 level is increased, while CD8^+^ T cells in such lesions without PD-1 expression are insensitive to PD-L1 interaction (104). In autoimmune thyroid diseases (AITDs), the PD-1/PD-L1 axis is activated but cannot suppress disease progression probably due to the limited PD-L1 expression *in vivo* (105). Notably, in individuals with RA, high levels of soluble PD-1 (sPD-1) can inhibit the PD-1/PD-L1 pathway, which appears to affect Treg maintenance (106). Studies have reported that the serum level of sPD-1 is correlated with the 28-joint disease activity score. In line with this finding, in celiac disease (CD) patients, excessive sPD-1 can inhibit the PD-1/PD-L1 pathway and cause aberrant T-cell proliferation (107). Additionally, recent studies have reported that a single-nucleotide polymorphism (SNP) in PDCD1 is associated with the development of autoimmune diseases (SLE, type 1 diabetes, progressive MS, Vogt-Koyanagi-Harada disease, Graves' disease, and RA), suggesting that PDCD1 may be involved in the maintenance of tolerance (20, 108). For instance, the SNP rs4143815 in PD-L1 is associated with T1DM and could be a new biomarker for predicting T1DM susceptibility (109, 110).

## Chronic Infection

Chronic infection is characterized by persistently high levels of antigen exposure, causing T cells to progressively lose effector functions and progress to exhaustion, similar to the state in cancer. Given its success in the treatment of cancer, anti-PD-1/PD-L1 therapy has potential in the treatment of severe chronic infections.

High PD-1 expression on CD8^+^ T cells was first reported in acute lymphocyte choriomeningitis virus (LCMV) infection. In addition, in infection with HIV, simian immunodeficiency virus, hepatitis B virus (HBV), and hepatitis C virus (HCV), PD-1 was observed to be expressed on virus-specific T cells (111–114). Studies have shown that several transcription factor binding sites regulate PD-1 expression on exhausted T cells. In the context of viral infection, binding of the transcription factor IFN regulatory factor 9 (IRF9) to the Pdcd1 promoter induces Pdcd1 transcription, which is also observed for the transcription factor forkhead box protein O 1 (FoxO1) (115, 116). Furthermore, during chronic infection, high expression of PD-1 can be induced by demethylation of the Pdcd1 promoter, which is observed in exhausted CD8^+^ T cells (117). Additionally, Alfei et al. (58) found that a new TOX molecule is correlated with PD-1 expression and can maintain the population of exhausted T cells. Metabolic processes play a vital role in the execution of the T-cell exhaustion program. Intriguingly, a recent report showed that PD-1 regulates several metabolic changes in the development of T-cell exhaustion (118). In chronic infection, PD-1 on T cells interacts with its ligands, which are found to be increased in monocytes, macrophages, and neutrophils, shutting down the T-cell response and leading to immunosuppression. CD4^+^ T cells expressing high PD-1 during HBV infection appeared to exhibit loss of helper T-cell function, and after treatment with PD-L1 blockade, the function of CD4^+^ T cells was partially restored (119). Combined blockade of the costimulatory molecule OX40 (CD134) and PD-L1 also improved the function of HBV-specific CD4^+^ T cells (120). Recently, Zhou (121) found that during LCMV and adenovirus infection, liver-resident NK (LrNK) cells expressing PD-L1 can control hepatic T-cell antiviral activity. In addition, blockade of PD-L1 can abolish the suppressive effect of LrNK cells on T cells (121). Notably, PD-L1-expressing neutrophils should attract attention for their importance in contributing to T-cell exhaustion because they are much more numerous than other immune cells. Moreover, recent studies have shown a link between PD-1/PD-L1 expression and clinical outcomes. In acute infection and untreated chronic infection, upregulation of PD-1 expression was linked to decreased effectiveness of CD4^+^ T-cell antiretroviral therapy (ART) (7, 122). PD-1 expression on CD8^+^ T cells after ART has been associated with impaired CD4^+^ T-cell immune reconstitution (123), microvascular diseases (124), and a reduced time to viral rebound after ART cessation (125). During the acute inflammatory phase of chronic HBV, elevated expression of PD-1/PD-L1 is required to offset the increasing positive costimulatory signals to prevent severe damage from an overvigorous immune response (7). Intriguingly, in *Listeria*-infected mice, PD-L1 binds to unknown receptors (not PD-1 or CD80) and is characterized as a costimulatory factor for antigen-specific CD8^+^ T cells (Table 1) (86).

PD-1/PD-L1 inhibitors can reinvigorate exhausted T cells. During LCMV infection, PD-1/PD-L1 pathway blockade can restore virus-specific CD8^+^ T-cell functions, promoting proliferation, cytokine production, and killing capacity. Additionally, PD-1/PD-L1 pathway blockade can reduce the viral load (111). Consistent with this finding, studies of HIV (a non-human primate model of simian immunodeficiency virus infection) and HBV/HCV (human infection with HBV/HCV and mouse and woodchuck models of HBV infection) infection showed similar results (112–114). In addition, studies have reported that PD-1 is required for maintaining T-cell exhaustion, but PD-1 inhibitors can enhance exhausted T-cell function. Therefore, the timing of PD-1/PD-L1 pathway modulation is critical (126). In addition to bolstering T-cell responses, treatment with anti-PD-1 antibody improved B-cell responses in a non-human primate model of HIV infection (127). In line with this finding, the combination of Tim-3 and PD-1 inhibitors reinvigorated exhausted CD8^+^ T cells and decreased the viral load in a mouse model of chronic infection (128). Intriguingly, in some cases, PD-1 blockade first restored virus-specific CD8^+^ T-cell function, but CD8^+^ T cells subsequently became exhausted again, displaying resistance to PD-1 immunotherapy (129). Studies have shown that during chronic infection, T cells that are sensitive to PD-1 inhibitors are the “progenitor-like” subset, and the less sensitive subset is the “terminally differentiated” subset (130–132). Progenitor-like T cells can transform into terminally differentiated T cells *via* proliferation, which may explain the above intriguing phenomenon (130, 131, 133).

Furthermore, PD-1/PD-L1 blockade may yield conflicting results in some infectious diseases. In influenza infection, *in vivo* blockade of PD-L1 was shown to reduce virus titers and increase the number of CD8^+^ T cells (Table 1) (83–85). In *M. tuberculosis* (Mtb) infection, PD-1 and PD-L1 expressions were enhanced in TB patients and Mtb-treated mice (134). PD-1 inhibitors can restore the effector functions (such as cytokine production and cytolytic activity) of NK cells and T cells (135), and PD-L1 blockade can increase the cytotoxicity of Mtb-specific CD8^+^ T cells to CD14^+^ cells from human tuberculous pleural effusion samples (136). However, the survival rate of PD-1-deficient mice challenged with Mtb infection is low (137, 138). Notably, high levels of Treg cells and mesenchymal stem cell infiltration may suppress specific T cells, leading to high mortality (Table 1) (138). In parallel, in a model of murine cerebral malaria, blocking PD-L1 induced inflammation and hemorrhage in the brain compared to its effects in untreated controls (Table 1) (87). However, PD-1 and LAG-3 blockade proved beneficial in murine malaria, with parasitemia reduction and improved CD4^+^ and CD8^+^ T-cell responses (88). In addition to the functions of PD-1/PD-L1, recent studies of malaria revealed a novel regulatory function of PD-L2. Expression of the PD-L2 protein on DCs that also express PD-L1 improved immune responses by inhibiting the PD-L1–PD-1 interaction. Moreover, administration of PD-L2 fused with the Fc region of immunoglobulin (PD-L2-Fc) to mice infected with malaria exerted a protective effect in this lethal infection (52).

Considering the success of PD-1/PD-L1 blockade in tumors, it is possible to promote enhanced immunity to chronic infection. Evaluation of the impact of PD-1/PD-L1 blockade in patients with chronic infection has been proposed. An anti-PD-L1 antibody (BMS-936559) underwent a phase II dose-escalation study in HIV infection with ART, the only trial in the setting of HIV infection without malignancy. Recently, this trial was ceased because of potential retinal toxicity, which was reported in a simultaneous macaque study (139). To date, anti-PD1 and anti-CTLA-4 antibodies both alone (NCT02595866) and in combination (NCT02408861) are in current clinical trials to evaluate their effects on HIV-associated malignancies (140). In addition, the anti-PD-1 antibody nivolumab, with and without an HBV vaccine, has been shown to suppress chronic HBV infection. A pilot study recently reported that in most virally suppressed HBV patients with negative HBeAg, treatment with nivolumab and the HBV vaccine (GS-4774) appeared to lead to HBsAg decline (7, 141).

## Sepsis

Sepsis, associated with high mortality in intensive care units, is characterized by severe immunosuppression after the first proinflammatory hours (142). Postmortem examination of deceased septic patients highlights key immunological defects that impair host immunity (143). Sepsis has immunosuppressive mechanisms similar to those of cancer. Given the potency of CTLA-4- and PD-1-specific antibodies in improving host immunity and increasing survival in cancer patients, these agents are expected to open a promising avenue to the development of novel medicines for sepsis (144).

It has been reported that in different animal models of sepsis, including bacterial, fungal, and burn sepsis, treatment with PD-1 or PD-L1 antibodies shows survival benefits (Table 1) (89, 91, 145, 146). Further studies found that PD-1- or PD-L1-deficient animals undergoing septic challenge also showed improved survival rates compared with WT animals (147). Importantly, in patients with sepsis and in septic animal models, the widespread expression of PD-1 and PD-L1 [on immune effector cells (monocytes, T lymphocytes, neutrophils, and NK cells), endothelial cells, and bronchial epithelial cells] suggests the potential ability of PD-1 and PD-L1 to be indicators of immunosuppression and therapeutic agents. Hotchkiss et al. found that in septic patients, PD-1 expression on T cells was elevated, which was linked to poor proliferation of T cells as well as a high risk of secondary nosocomial infections and mortality (91). In addition, in septic shock patients, increased expression of PD-L1 in monocytes is correlated with 28-day mortality (Table 1) (92), and the abundance of PD-L1-expressing neutrophils is a marker of disease severity and predicts prognosis in sepsis (Table 1) (90, 148).

The beneficial effects of anti-PD1 and anti-PD-L1 therapy have been confirmed, but the mechanism underlying these effects is unclear. Given the complexity of the cellular targets and activities of PD-1 and PD-L1, different mechanisms may be involved. Generally, PD-1 is expressed on innate immune cells and binds to PD-L1. For example, binding of PD-L1 to PD-1 expressed on DCs leads to an increase in the apoptosis of DCs and a decrease in the levels of proinflammatory cytokines (149). Peripheral blood monocytes from patients with sepsis showed increased levels of PD-L1, and binding with PD-1 reduced cell survival and function, while treatment with anti-PD-1 antibody restored the production of the key cytokines IFN-γ and IL-2 by monocytes (Table 1) (93). However, recent reports indicate that PD-1 and PD-L1 function very differently and possibly separately. For example, macrophages from septic animals retain increased PD-1 expression in the absence of PD-L1 (147). In addition, Huang et al. (150) demonstrated that during sepsis, peritoneal macrophages upregulate PD-1 expression, and the ability of macrophages to phagocytose and clear bacteria is PD-1-dependent; PD-L1 has a minor role in this process. Notably, the site at which PD-1 and PD-L are expressed may affect their functions. Studies show that in animal models of sepsis, high expression of PD-L1 in the liver can protect the liver from damage (151). A study in a mouse model of hemorrhagic shock/CLP-induced indirect acute lung injury (iALI) found that PD-L1 can modulate the ability of Tregs to suppress iALI, but this ability was abolished when wild-type (WT) Tregs were adoptively transferred to PD-L1^−/−^ recipient mice (152). Another post-mortem study showed detectable PD-L1 expression in the lungs of septic patients. Septic patients are often susceptible to secondary lung infections, which may contribute to this increase in PD-L1 expression, thereby suppressing T-cell function (143, 153).

Furthermore, immunotherapeutic approaches that use anti-PD-1/PD-L1 antibodies warrant further investigation. To date, some anti-PD-1 and anti-PD-L1 antibodies, including nivolumab (anti-PD-1) and BMS-936559 (anti-PD-L1), are in current clinical trials to evaluate their effects on severe sepsis/septic shock (154).

## Others

The immunoregulatory role of the PD-1/PD-L pathway in other disorders, including ischemia–reperfusion injury (IRI), stroke, Alzheimer's disease (AD), and pain, has been reported.

Studies in mouse models with blockade of PD-L1 and/or PD-L2 or knockout of PD-Ls confirmed that PD-L1 and PD-L2 play a protective role in kidney IRI (155). Furthermore, when recipient mice were treated with inhibitors of either PD-L1 or PD-L2 before adoptive transfer of Tregs, the protective effect of Treg transfer was abolished, suggesting that the therapeutic mechanisms of adoptive Treg transfer are attributed to ligand inhibition. This phenomenon was also observed in a model of acute liver IRI, in which blocking PD-L1 increased liver damage and treatment with a PD-L1 Ig fusion protein reduced liver inflammation (156).

The PD-1/PD-L pathway has also been studied in post-stroke inflammation. Interestingly, in ischemic brain injury, peripheral B cells inhibit the activation of effector T cells, resident microglia, and infiltrated macrophages *via* the PD-1/PD-L1 pathway, thereby reducing inflammation (157). In an experimental model of stroke, 4 days after transient middle cerebral artery occlusion, PD-L1 and PD-L2 expression was increased on B cells in the central nervous system (CNS), blood, and spleen (158). In addition, in the acute phase after shock, the expression of PD-L1 is enhanced on Tregs, suppressing the release of matrix metalloproteinase-9 (MMP-9) from neutrophils, thereby alleviating damage to the blood–brain barrier (159). In contrast, knockout of PD-L1 or PD-L2 in mice exhibits an adverse effect on stroke outcomes and elevates inflammation in the post-stroke period. Notably, PD-L1 also plays a role in enhancing the severity of stoke (160). The current study demonstrated that the enhanced post-stroke inflammation and brain injury are due to the decreased number of CD8^+^CD12^+^ suppressor T cells in the CNS, which are PD-L1-dependent. The studies mentioned above show the different aspects of the PD-1/PD-L pathway, which reflects the dualistic nature of the immune system.

Patients with mild cognitive impairment or a diagnosis of AD express lower levels of PD-1 on CD4^+^ T cells and lower levels of PD-L1 on CD14^+^ macrophages/monocytes (161). With the disruption of the PD-1/PD-L1 pathway, the release of IL-10 is decreased (162). It is known that IL-10 can ameliorate pathology in animal models of AD, indicating that the remission of AD is, at least in part, due to the PD-1/PD-L1 pathway (162). However, the molecular and cellular mechanisms of the PD-1/PD-L1 interaction in AD and the influence of this interaction on immune cells in the CNS need to be further studied.

PD-L1 can also serve as an endogenous pain inhibitor and a neuromodulator. It can also be expressed in the spinal cord, dorsal root ganglia (DRG), nerves, and skin (163). In naive mice, PD-L1 can exert an analgesic effect by interacting with PD-1 and, conversely, blockade of either PD-1 or PD-L1 elicits mechanical allodynia. Moreover, PD-L1, as a unique neuromodulator, can suppress spinal cord synaptic transmission in the pain circuit. Mechanistically, PD-1 is expressed in nociceptive neurons in the DRG, and ligand binding to PD-1 leads to SHP-1 phosphorylation and triggers hyperpolarization of TREK2 K+ channels. Nociceptive neurons have been inhibited by PD-L1, and we anticipate a future painkiller based on PD-L1.

## Resistance to and Toxicity of PD-1/PD-L1 Inhibitors

PD-1/PD-L1 inhibitors are a major advance, but they are not without drawbacks. As PD-1 and PD-L1 play a crucial role in peripheral immune tolerance, blockade of this immune checkpoint could lead to novel immune-related adverse events (irAEs). The most commonly observed irAEs include colitis, hepatitis, myositis, pneumonitis, endocrinopathies, kidney injury, and skin toxicities (164). Serous diarrhea and colitis often occur under combination therapy with ipilimumab and nivolumab (15%) but are less frequently observed in patients receiving anti-PD-1 monotherapy (1–4%) (165–167). Hepatitis occurrence varies with the immune checkpoint inhibitor (ICI) agent. Hepatitis occurred in up to 7% of individuals treated with anti-PD-1 monotherapy and in up to 33% of individuals treated with anti-PD-1 in combination with anti-CTLA-4 (168, 169). Usually, this hepatitis presents as asymptomatic elevated transaminase levels with or without elevation of the bilirubin concentration (167). Cardiac irAEs due to PD-1 axis inhibitors occur in <1% of treated individuals. However, high mortality rates have drawn attention to these events. Based on pharmacovigilance studies, compared with nivolumab monotherapy, treatment with a combination of ipilimumab and nivolumab resulted in an increased incidence of myocarditis (0.27 vs. 0.06%) and increased severity of myocarditis (0.17 vs. 0.01%) (170). With anti-PD-1/PD-L1 monotherapy, the incidence of pneumonitis is ~5%, and in those with combination immunotherapy, the incidence can be up to 10%. The onset of pneumonitis is often earlier and more severe in patients with NSCLC than in patients with other malignancies treated with PD-1 inhibitors (164, 165). Endocrinopathy irAEs due to PD-1 axis inhibitors include hypophysitis, thyroiditis, hypothyroidism, hyperthyroidism, and T1DM (171). Unlike the majority of irAEs, endocrinopathies are usually irreversible (165). Under anti-PD-1 monotherapy, patients often experience worsening renal function (13–22%) (172). Patients treated with ICI combination therapy were more susceptible to acute kidney injury (AKI) than those who received ipilimumab, nivolumab, or pembrolizumab monotherapy (173). Under treatment with anti-PD-1 agents, 30–40% of patients experience skin toxicities (174). The most common skin toxicities are lichenoid reactions, vitiligo, pruritus, and eczema (175). Intriguingly, vitiligo was observed only in patients with metastatic melanoma and not in patients with any other tumors (176). In addition to the above irAEs, some rare toxicities mediated by checkpoint inhibitors are also observed, such as neurological, ocular, and hematological toxicities (172). In clinical trials, there is no consensus definition to guide reporting of irAEs because irAEs are a novel presentation of toxicity from ICI treatment. Therefore, it is challenging for the physician to give an accurate report of the occurrence of irAEs. It has been reported that irAEs may be linked to dysregulated immune cell subpopulations. For instance, PD-1 blockade in viral infections or cancer increases the risk of cardiovascular complications, which is at least partially due to the effect of PD-1 on proatherogenic T cells (11). Patients (mainly those with melanoma and NSCLC) treated with PD-1/PD-L1 blockade were diagnosed with checkpoint inhibitor pneumonitis showing increased bronchoalveolar lavage (BAL) lymphocytosis, especially involving central memory T cells, and decreased expression of CTLA-4 and PD-1 in BAL Tregs (177). Moreover, the extent of autoantibodies could also lead to immune-related adverse events. In a report of thyroid disorders, these events occurred when patients with antithyroid antibodies received anti-PD-1 therapy, suggesting that anti-PD-1 treatment may regulate preexisting antithyroid antibodies (178). Similar T-cell clones were found in normal and tumor tissue, suggesting that T-cell cross-reactivity may result in injury to normal tissue. This phenomenon has been observed in patients with melanoma treated with an ICI, which led to vitiligo (179). In addition to the adverse effects of PD-1/PD-L1 inhibitors, resistance to PD-1/PD-L1 inhibitors is observed in some cancer patients, which is reported to be partially associated with an abnormal gut microbiome composition. Routy et al. (180) used fecal microbiota transplantation (avatar mice) to determine the relationship between the anticancer efficacy of PD-1 blockade and the dominance of commensal species and found that the intestinal microbiota influences the outcome of PD-1 blockade in mice and patients. Additionally, Chen et al. (181) found that tumor cells expressing CD38, which regulates the adenosine production pathway, escape from checkpoint inhibitor-mediated immune attack. In patients with advanced melanoma, resistance to PD-1 blockade therapy was reported to be linked to genetic aberrations in the cyclin D–cyclin-dependent kinase 4 (CDK4) pathway (182). Moreover, anti-PD-1 antibody binding to Fc receptors expressed on tumor-associated macrophages can partially be removed from tumors, which may reduce the effect of PD-1 antibodies (183). Furthermore, with high levels of exosomal PD-L1, T cells may no longer be reinvigorated *via* anti-PD-1 treatment, shedding light on the possible cause of resistance to PD-1 inhibitor therapy (184). Notably, in some tumors, such as melanoma, PD-L1 can mask pain; conversely, suppression of PD-L1 or PD-1 induced pain in tumor-bearing mice. In patients diagnosed with cancer, the pain level should be assessed before, during, and after immunotherapy (163). Recent reports have shown that in certain cancer patients, treatment with anti-PD-1 mAb can promote rapid disease progression, which is called hyperprogressive disease (HPD). Kamada et al. (185) found that in patients and mice with advanced gastric cancer, anti-PD-1 mAb increased the proliferation of FoxP3^high^CD45RA^−^CD4^+^PD-1^+^T cells [effector Treg (eTreg) cells], which could suppress antitumor immunity. In addition, depletion of these Treg cells could be beneficial for preventing HPD in the setting of PD-1 blockade therapy (185). Therefore, the use of PD-1 and PD-L1 inhibitors should be considered when diagnosing and treating diseases.

## Concluding Remarks

**Figure 2:**
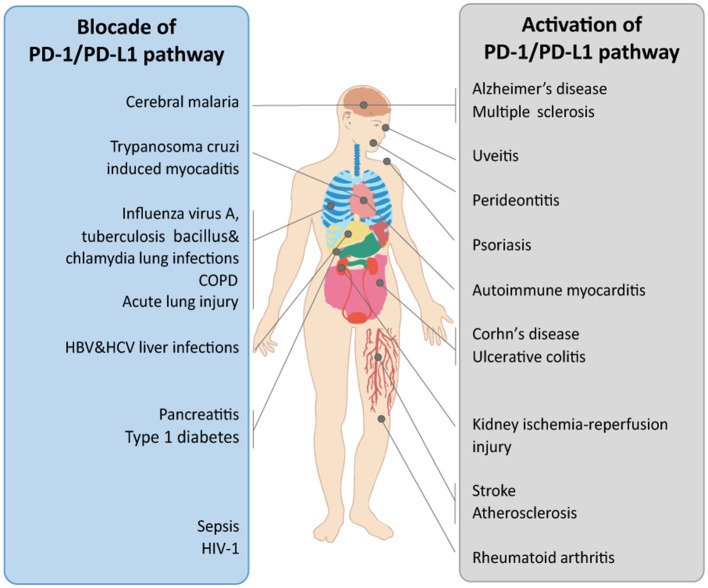
The therapeutic potential of the PD-1/PD-L1 pathway in organ-related diseases. The PD-1/PD-L1 pathway is implicated in multiple diseases. In different diseases, the PD-1/PD-L1 pathway shows diverse functions. The therapeutic potential of the PD-1/PD-L1 pathway when treated *via* blockade or activation in diverse organ-related diseases is shown. COPD, chronic obstructive pulmonary disease.

The PD-1/PD-L signaling pathway plays a pivotal role in peripheral immune tolerance, which prevents inappropriate immune responses under physiological conditions. However, in infection or cancer, the alternative role of this pathway leading to immune suppression could cause serious issues. Thus, the functions of PD-1/PD-L in different diseases should be considered. The discrepant roles of PD-1/PD-L pathway observed in various diseases may be attributed to the different phases of disease, the dual effects of the PD-1/PD-L axis (Figure 2), the specific cells expressing PD-1/PD-Ls, and the unknown receptors to which PD-L1 binds. Moreover, the influence of the gut microbiome and the potential side effects should always be considered. As the mechanisms by which PD-1/PD-L1 blockade therapy functions and the mechanisms of resistance and toxicities are elucidated, the development of more precise treatments for different diseases is anticipated. Furthermore, the implementation of these treatments may inform researchers' and clinicians' choice of the appropriate combinatorial approaches to produce more rapid results.

## Author Contributions

All authors listed have made a substantial, direct and intellectual contribution to the work, and approved it for publication.

### Conflict of Interest

The authors declare that the research was conducted in the absence of any commercial or financial relationships that could be construed as a potential conflict of interest.
